# Physical Activity in Older Mexican Americans Living in Two Cities on the U.S.-Mexico Border

**DOI:** 10.3390/ijerph15091820

**Published:** 2018-08-23

**Authors:** Gerardo Vasquez, Jennifer Salinas, Jennifer Molokwu, Gurjeet Shokar, Silvia Flores-Luevano, Adam Alomari, Navkiran K. Shokar

**Affiliations:** 1 Department of Family Medicine, Paul L. Foster School of Medicine, Texas Tech University Health Sciences Center El Paso, El Paso, TX 79905, USA; Gerardo.Vasquez@ttuhsc.edu (G.V.); jennifer.molokwu@ttuhsc.edu (J.M.); gurjeet.shokar@ttuhsc.edu (G.S.); adam.alomari@ttuhsc.edu (A.A.); Navkiran.Shokar@ttuhsc.edu (N.K.S.); 2 Instituto Mexicano del Seguro Social, Ciudad de Mexico 06600, Mexico; silflor33@gmail.com

**Keywords:** physical activity, Hispanics, context

## Abstract

Background: There is limited information on physical activity in marginalized older populations like that on the U.S.-Mexico border. This study aims to understand physical activity engagement among older Hispanics residing in two U.S.-Mexico Border counties. Methods: The International Physical Activity Questionnaire (IPAQ) was used to measure physical activity in El Paso and Cameron County, Texas. Physical activity levels were reported for vigorous, moderate, and walking met/mins. Adjusted and unadjusted modeling was conducted to determine county differences and sociodemographic covariates. Results: There were 784 participants and 92.9% were less than 65 years of age. El Paso participants reported a significantly greater natural log met/mins of vigorous (*β* = 1.34, *p* = 0.000) and walking (*β* = 0.331, *p* = 0.006). Significant sociodemographic covariates in El Paso for vigorous met/mins were gender (females *β* = −1.20, *p* = 0.003), having a regular doctor (*β* = −0.779, *p* = 0.029), and acculturation (*β* = 0.513, *p* = 0.019). Significant associations in Cameron County were having a regular doctor (*β* = −1.03, *p* = 0.000) and fair/poor health status (*β* = −0.475, *p* = 0.001). Conclusion: Level of physical activity may differ in older Hispanics by urban context on the U.S.-Mexico border. Future physical activity programs to promote physical activity should take context into consideration.

## 1. Introduction

There is compelling evidence that an insufficient amount of physical activity is associated with an increased risk of chronic metabolic, cardiovascular disease, and several forms of cancer [1,2,3,4]. The scientific evidence of the benefits of regular physical activity is indisputable, and the benefits far outweighs the risks for most adults. Current Centers for Disease Control and Prevention (CDC) guidelines recommend that older adults engage in either moderately intensive cardiorespiratory physical activity training (brisk walking) for at least 30 min five or more days per week (150 min per week) or vigorous cardiorespiratory physical activity training (jogging, running, aerobic dance) for 20 min or more per day for three days or more per week [5]. Older adults with medical comorbidities can safely participate in physical activity with the advice of their health care professional, and regular physical activity provides substantial benefits in reducing chronic disease symptoms and comorbidities. [5]. Furthermore, engaging in these activities can help achieve weight loss and maintain a lower body mass index (BMI) [6].

Despite the noted benefits, only 21% of U.S. adults meet the CDC guidelines [7]. In addition, disparities by race/ethnicity exist, with 23% of non-Hispanic whites achieving national standards compared to 16% of Hispanics [7]. Epidemiological information on physical activity among older age Hispanic subgroups, in general, is limited [8]. Most physical activity studies have used large datasets, taken place in subpopulations in the northeastern United States, or as part of intervention studies [9,10,11,12,13,14,15,16,17,18,19], ignoring nuances in subcommunities like that of the U.S.-Mexico border region. There is growing evidence that geographic location and context is an important predictor of physical activity engagement [20,21,22]. The consensus is that places that people reside in are highly predictive of physical activity engagement [23,24,25,26] and residents of places with greater amenities to be active are more likely to meet recommended guidelines to be active [27,28].

The U.S.-Mexico border region is a diverse ecology stretching from the Pacific Ocean in California to the Gulf Coast of Texas [29]. County sizes along the border vary from 7156 in Presidio Texas to over 3 million in San Diego California. Because of this diversity, context of factors associated with physical activity on the U.S.-Mexico border must be taken into consideration to better understand specific barriers or differences that may exist in this region. This area is particularly important to understand the physical activity of Hispanics, since the largest concentration of Hispanics, primarily Mexican Americans, live on or near the U.S.-Mexico border [30]. In this study, we make use of a unique dataset from two contrasting counties on the U.S.-Mexico border: El Paso and Cameron Counties. While both counties are predominantly of Mexican origin (El Paso 82.8% and Cameron 89.7%) [31], they offer an opportunity to understand how physical activity engagement among older Mexican Americans may vary by context along the U.S.-Mexico border. While both are on the U.S.-Mexico border, the socioeconomic landscapes of these two counties are different. El Paso has over 800,000 people residing within the county, compared to about 40,000 in Cameron [32]. Therefore, while El Paso is considered an urban county, Cameron is deemed rural. The simple differences between urban/rural population statuses also translates into stark socioeconomic differences. For example, 78.0% of adults in El Paso have a high school diploma or more, compared to only 64.1% of adults in Cameron County. Additionally, 20.9% of persons residing in El Paso live in poverty compared to 33.7% in Cameron County [31]. Recognizing differences in physical activity patterns among older adult Hispanic subgroups has important implications for behavioral health research and for the planning of culturally and age-sensitive health promotion interventions for the U.S.-Mexico border region [33].

There is limited information on how physical activity in older Mexican Americans may differ depending on the context in which they live, particularly for those who reside on the U.S.-Mexico border. Therefore, the objective of this study is to contrast physical activity patterns among older Mexican Americans residing in two U.S.-Mexico border counties. In this study, we compared two samples of older Mexican Americans surveyed for the evaluation of a colon cancer outreach and education program in El Paso and Cameron Counties, Texas. We expected that being larger and more urban, physical activity levels would be greater in those participants residing in El Paso. We also expected that any differences between the two counties would be explained in part by socioeconomic factors.

## 2. Materials and Methods

### 2.1. Participants

This is a secondary data analysis of cross-sectional baseline data obtained from 784 participants as part of a study that evaluated a colorectal cancer (CRC) screening intervention. Original data for this analysis were collected between March 2012 and August 2015 as part of an evidence-based program, Against Colorectal Cancer in Our Neighborhoods (ACCION), designed to reduce the burden of CRC in El Paso County [34]. Prior IRB approval was obtained. Participants in the study were recruited from community and clinical sites in two U.S.-Mexico Border counties: El Paso and Cameron (the comparison group). Eligibility criteria for inclusion were age of 50–75 years, self-reported Texas address, due for colorectal cancer screening at time of recruitment, and no blood in the stool for the previous three months.

### 2.2. Measures

#### Dependent Variable

International Physical Activity Questionnaire (IPAQ). We made use of the IPAQ to measure physical activity across an inclusive set of activities, such as leisure time, domestic, work-related, and transport-related movements [35]. The IPAQ (short version) was used to measure vigorous activity (hard physical effort that makes you breathe much harder than normal), moderate activity (moderate physical effort that makes you breathe somewhat harder than normal), and walking (including at work, home, solely for recreation, sport, physical activity, or leisure) [36,37]. Participants were asked to report the number of days per week and the number of hours/minutes per day these activities were performed. The IPAQ has a reliability of 0.80 and validity of 0.30 internationally [37].

Using the energy requirement criteria determined for metabolic equivalents (METs) in physical activity testing, all responses for activity measures were converted from hours into minutes to produce a continuous score. MET is the ratio of a person’s working metabolic rate relative to the resting metabolic rate. One MET is defined as the energy cost of sitting quietly and is equivalent to a caloric consumption of 1 kcal/kg/h [36]. For each specific type of activity, the frequency (measured in days per week) and duration (time per day) were recorded; vigorous activity was defined as 8 METs, moderate as 4 METs, and walking as 3.3 METs. Each form of activity was assigned a formula in order to determine a score for the participant calculated by multiplying its METs by frequency (days per week) and by duration (time per minutes). The subject’s overall physical activity was calculated by using the total physical activity MET-minutes/week summing (walking + moderate + vigorous) MET-min/week scores. Listed below are the designated formulas for each activity [37]:

Walking MET-min/week = 3.3 × walking minutes × walking days.

Moderate MET-min/week = 4.0 × moderate-intensity activity minutes × moderate days

Vigorous MET-min/week = 8.0 × vigorous-intensity activity minutes × vigorous intensity days.

We made use of the three categories—vigorous, moderate, and walking met/mins—for our analysis.

### 2.3. Covariates

Demographic variables measured include gender, age, birth country (US/Mexico/other), recruitment location (El Paso vs. Cameron County), education (high school or less/more than high school), income (less than $10,000/$10,000–$20,000, more than $20,000), acculturation (continuous for Table 1, Table 2 and Table 3; categorical for Table 2; low-mostly Spanish speaker vs. high-mostly English speaker) self-reported health status (excellent, very good, good, fair, poor), smoking status (current, past, never), marital status (married/cohabitation status yes/no), and having a regular doctor (yes/no).

### 2.4. Analysis

Statistical analysis was conducted using Stata MP 14 [38]. Continuous variables were described using mean, standard deviation, and frequency, and percentages were used to describe categorical variables. We used Spearman’s correlation coefficient test to evaluate the association between IPAQ scores and continuous demographic variables. Pearson’s chi-squared test was used to calculate scores between categorical demographic characteristics and the association with physical activity by county. *p*-values less than 5% were considered significant results. Adjusted multivariable ordinary least squares (OLS) regression analysis was conducted to assess differences by site for each IPAQ outcome. Because of significant skewness in all three IPAQ outcomes, natural log transformations were used in this analysis.

## 3. Results

The majority of the participants were over 65 years of age (92.9%), were female (78.6%), and born in Mexico (90.3%) (see Table 1). Additionally, most had less than a high school level of education (77.8%), made less than $10,000 per year, and spoke mostly Spanish (average score 1.31 out of 5). Most were married (63.2%), rated their health as excellent, very good, or good (55.2%), never smoked (59.4%), and did not have a regular doctor (61.8%). Significant differences between El Paso and Cameron Counties were gender, educational attainment, and health status. When compared to Cameron County, a greater proportion in El Paso were male (25.3% vs. 15.6%, *p*-value 0.001), a lower proportion with high school or greater education (74.3% vs. 84.5%, *p*-value 0.000), and rated their health as excellent/very good or good (58.0% vs. 50.9%, *p*-value 0.051). Finally, near significant differences were observed for having a regular doctor (El Paso 37.3% vs. Cameron 44.4%, *p* = 0.081).

Table 2 presents the unadjusted associations between demographic variables and the IPAQ outcomes by county. Only significant covariates from the univariate analysis in Table 1 were used. Cameron County participants reported on average less total vigorous activity than in El Paso (283.6 vs. 1641.1 met/mins). Significant differences were observed in El Paso by gender, whereas men had on average higher met/mins of vigorous (3325.8 vs. 1071.5, *p* < 0.001), moderate (1022.3 vs. 482.1, *p* < 0.01), and walking (1039.6 vs. 528.6, *p* < 0.001) compared to women. In El Paso, participants reported higher average met/mins of walking in the unadjusted analysis if they had a greater than high school educational attainment (888.3 vs. 573.4, *p* < 0.05), speak more English than Spanish (1338.5 vs. 576.2, *p* < 0.05), are current smokers (1040.9 vs. 470.1, *p* < 0.05), and did not have a regular doctor (805.9 vs. 424.4, *p* < 0.01). In Cameron County, participants who reported not having a regular doctor reported higher average met/mins of vigorous activity (386.7 vs. 148.6, *p* < 0.05) but lower average walking met/mins (185.3 vs. 301.8, *p* < 0.01). Also, in Cameron County, participants who reported their health as excellent, very good, or good reported higher average met/mins of walking (284.5 vs. 189.9, *p* < 0.05).

Table 3 presents the results stratified by county and the between-county comparison OLS regression results for the natural log transformation IPAQ outcomes. We ran two models. In the first model, we compared El Paso to Cameron County, adjusting for gender, education, health status, regular doctor, smoking history, and acculturation. In this analysis, participants in El Paso reported 3.8 times higher average natural log IPAQ met/mins of vigorous activity (*β* = 1.34, *p* = 0.000) and 1.39 times higher for walking (*β* = 0.331, *p* = 0.006). We then ran separate models for El Paso and Cameron Counties to determine significant within-county covariates for our IPAQ outcomes. Females in El Paso on average engaged in 26.4% (*β* = 1.33, *p* = 0.001) less vigorous physical activity than men. Participants who had a regular doctor had a 45.8% (*β* = −0.780, *p* = 0.027) lower average vigorous met/mins in El Paso and 64% lower in Cameron County (*β* = −1.03, *p* = 0.000). Additionally, El Paso participants had on average a 167% increase in vigorous activity for each point increase in acculturation (*β* = 0.513, *p* = 0.019). In Cameron County, health status was associated with 38% less met/mins of walking (*β* = −0.475, *p* = 0.001) for participants rating their health as fair or poor.

## 4. Discussion

This study’s findings revealed that on the U.S.-Mexico border, there were significant differences by county. Participants in El Paso were nearly three times more active when compared to Cameron County participants. Our findings were consistent with the leisure time physical inactivity prevalence documented by national surveillance programs like the Behavioral Risk Factor Surveillance Survey (BRFSS) (El Paso 27.0% vs. Cameron County 36.8%) [39], adding to the narrative that along the U.S.-Mexico border, there are contextual differences in physical activity engagement. Socioeconomic predictors did vary by context, although they did not fully explain the differences between El Paso and Cameron Counties for vigorous or walking activity.

Even adjusting for several individual factors, differences continued to exist for both vigorous and walking activity by county. These findings may suggest that there are unmeasured attributes of each location that may be responsible for these differences. Despite both being on the U.S.-Mexico border, El Paso is a much larger county than Cameron County and, as such, may have more amenities for physical activity, such as better access to parks, trails, and bike paths, which may in turn be responsible for the higher levels of physical activity. In the few studies that have compared metropolitan areas, size and socioeconomic context have been identified as important factors in physical activity amenities [40,41]. Although the exact mechanisms are not well known, individual and community resources may limit or enable real or perceived options for activity. More research is needed to better understand contextual variations along the border, such as rural/urban or built environment policies that may contribute to differentials in physical activity engagement by county in this region.

Many studies have established socioeconomic correlations to physical activity resources at a neighborhood level [42,43,44], but few have done so by metropolitan area. In this study, participants in Cameron County that had a regular doctor or fair/poor health engaged in less physical activity. Socioeconomic characteristics that were associated with physical activity in El Paso were gender, having a regular doctor, and acculturation. Other studies have shown similar levels of physical activity and socioeconomic predictors in Cameron County [34], but no study has compared the same socioeconomic characteristics to different contexts in the United States or on the border [45,46]. Participants who identified as having a regular medical provider may be less active because of existing health conditions (e.g., fair/poor health status) in Cameron County. Healthcare access is limited in Cameron County, and it is recognized as medically underserved [47]. Less access, in this context, may translate into poorer overall health and less capacity to engage in physical activity.

In the context of El Paso, acculturation and male gender were predictive of more physical activity engagement, which is consistent with the literature from other contexts [48]. Men have been well documented to engage in physical activity more often than women [49,50], although few studies have compared gender differences in Hispanic groups [51]. Acculturation has been well studied in the Hispanic health literature [52,53]. In general, greater acculturation to the United States is associated with more physical activity but a less healthy diet [54,55,56]. El Paso, due to its size and fewer socioeconomic burdens, may be more in line with other large U.S. cities, despite being on the border, and less similar to more rural, less developed border areas like Cameron County, resulting in similar predictors to other large metropolitan areas. More research on border context comparisons are needed to disentangle what is truly border from rural/urban or other factors that may influence physical activity that may distinguish one are from the other.

There are a number of limitations to this study. Most notably, the data was collected from only El Paso and Cameron Counties, and inferences cannot be made to other areas of the U.S.-Mexico border or other Hispanic subpopulations. Additionally, since this data is cross sectional, we were unable to establish any causal relationships. Furthermore, while the IPAQ is widely used, it is a subjective measure of reporting physical activity which could over- or underestimate actual levels of activity. Finally, participants in the ACCION program were recruited because they were overdue for colon cancer screening and uninsured, so the sample represents a subpopulation of those living on the U.S.-Mexico border.

The strengths of this study include the large sample of older adults, the comparison of two metropolitan areas on the border, and the use of a widely used, validated measure of physical activity. Future studies should investigate the age-specific benefits of physical activity in older Hispanic populations living in different contexts in the U.S.-Mexico border region and other areas across the U.S. In addition, clinic-based interventions using the primary care physician (PCP) to counsel patients about age-appropriate physical activity levels may be an effective approach to increasing physical activity levels in this population. There is limited physical activity education currently being conducted in clinical settings, particularly among Mexican American populations [57,58]. Other areas where further investigation is needed is a comparison of different Hispanic subgroups and variations in levels of physical activity that may be impacted by context.

## 5. Conclusions

Overall, physical activity levels are much lower on U.S.-Mexico border than what has been documented nationally. In addition, Cameron County, a more socioeconomically burdened county on the U.S.-Mexico border, may be at increased risk for obesity and associated chronic diseases due to high levels of physical inactivity established in this study. Physical activity is a low-cost solution to a growing epidemic of chronic disease in this region, and finding ways to increase activity will in the long run reverse current trends and improve the wellbeing of the older population of Mexican Americans.

## Figures and Tables

**Table 1:** Demographic characteristics and physical activity levels in the Against Colorectal Cancer in Our Neighborhoods (ACCION) sample by county of residence.

	El Paso County	Cameron County	Total	*p*-Value
**Variables**				
**Gender**				
Male	118 (25.3)	49 (15.6)	167 (21.4)	0.001
Female	349 (74.7)	265 (84.4)	614 (78.6)	
**Age (years)**				
<65	434 (92.9)	295 (93.1)	729 (92.9)	0.946
65+	33 (7.1)	22 (6.9)	55 (7.1)	
**Country of Birth**				
Mexico	421 (90.3)	281 (88.6)	702 (90.3)	0.715
USA	42 (9.0)	33 (10.4)	75 (9.6)	
Other	3 (0.06)	3 (0.09)	6 (0.77)	
**Education All**				
<High School	342 (74.3)	268 (84.5)	610 (77.8)	0.000
>High School	125 (26.7)	49 (15.5)	174 (22.2)	
**Income**				
Less than $10,000	194 (63.2)	161 (63.9)	355 (63.5)	0.258
10,000 to less than 25,000	101 (32.9)	74 (29.4)	175 (31.3)	
25,000 or more	12 (3.9)	17 (6.7)	29 (5.2)	
Acculturation	1.39 (0.807)	1.19 (0.582)	1.31 (0.731)	0.0001
**Health Status**				
Poor/Fair	196 (42.0)	155 (49.1)	351 (44.8)	0.051
Excellent/Good/Very Good	271 (58.0)	161 (50.9)	432 (55.2)	
**Smoking History**				0.043
Never	258 (56.1)	203 (64.2)	461 (59.4)	
Past Smoker	144 (31.3)	87 (27.5)	231 (29.8)	
Current Smoker	58 (12.6)	26 (8.2)	58 (12.6)	
**Married/Cohabitating**				
Yes	285 (61.2)	209 (66.1)	494 (63.2)	0.157
No	181 (38.8)	107 (33.9)	288 (36.8)	
**Regular Doctor**				
Yes	174 (37.3)	140 (44.4)	314 (40.7)	0.081
No	282 (60.5)	175 (55.6)	457 (59.3)	

**Table 2:** The unadjusted association between demographic variables and the International Physical Activity Questionnaire (IPAQ) outcomes (met/min per week).

Variables	Vigorous		Moderate		Walking	
	El Paso	Cameron County	El Paso	Cameron County	El Paso	Cameron County
**Total (mean ± s.d.)**	1641.1 (4961.1)	283.6 *** (949.6)	618.6 (1920.2)	188.3 (685.6)	657.7 (1492.9)	235.6 (382.0)
**Gender (mean ± s.d.)**						
Male	3325.8 *** (7494.8)	377.1 (1744.5)	1022.3 ** (2746.3)	130.6 (401.6)	1039.6 *** (2235.4)	178.1 (370.3)
Female	1071.5 (3575.5)	264.2 (719.9)	482.1 (1525.7)	201.1 (729.5)	528.6 (1112.9)	245.1 (382.6)
**Education (mean ± s.d.)**						
<High School	1648.9 (5229.4)	298.8 (1004.5)	749.4 (1884.5)	133.9 (294.0)	573.4 * (1394.1)	224.6 (381.5)
>High School	1619.9 (4158.1)	200.8 (561.5)	570.8 (1933.6)	198.2 (735.0)	888.3 (1720.2)	295.7 (382.6)
**Acculturation (mean + s.d.)**						
More English	1683.1 (3640.6)	192 (607.2)	1338.5 * (3016.3)	300 (546.3)	1252.9 (1928.9)	64.4 (140.1)
More Spanish	1638.7 (5031.2)	286.6 (959.2)	576.2 (1831.9)	184.6 (690.1)	622.6 (1458.5)	241.2 (386.1)
**Health Status (mean ± s.d.)**						
Poor/Fair	1410.4 (4820.0)	229.6 (1023.6)	597.4 (2032.7)	212.4 (864.8)	623.3 * (1567.4)	189.9 * (450.7)
Excellent/Very Good/Good	1960.2 (5145.2)	341.7 (868.7)	647.9 (1757.5)	164.4 (431.4)	705.2 (1438.8)	284.5 (296.5)
**Smoking History (mean + s.d.)**						
Never	1203.2 (3869.1)	239.0 (699.7)	470.1 (1660.9)	201.7 (786.2)	582.9 (1186.7)	258.2 (405.4)
Past Smoker	2113.9 (6250.6)	459.3 (1444.8)	715.8 (1905.4)	167.1 (473.3)	752.2 (1841.9)	201.9 (345.1)
Current Smoker	2470.2 (5726.0)	55.4 (282.4)	1040.9 * (2846.4)	161.5 (414.2)	789.1 (1796.7)	142.1 (257.8)
**Regular Doctor (mean ± s.d.)**						
Yes	1197.1 (4307)	148.6 (649.3)	459.9 (1694.7)	130.4 (319.1)	424.4 ** (828.7)	301.8 (433.3)
No	1949.0 (5391.9)	386.7 * (1125.7)	728.8 (2072.9)	236.7 (875.8)	805.9 (1785.5)	185.3 (329.1)

**Table 3:** Adjusted regression results for natural log IPAQ outcomes El Paso vs. Cameron Counties.

Variables	lnVigorous		lnModerate		lnWalk	
	El Paso	Cameron County	El Paso	Cameron County	El Paso	Cameron County
**Gender (ref. cat. = female)**	−1.20 (0.003)	0.584 (0.164)	−0.374 (0.132)	0.596 (0.157)	−0.252 (0.138)	−0.141 (0.544)
**Education (ref. cat. ≥ High School)**	0.073 (0.858)	−0.308 (0.461)	0.370 (0.169)	−0.068 (0.807)	0.068 (0.686)	−0.012 (0.947)
**Health Status (ref. cat. = Fair/Poor)**	−0.651 (0.066)	−0.259 (0.373)	−0.169 (0.467)	0.168 (0.406)	0.161 (0.299)	−0.475 (0.001)
**Regular Doctor (ref. cat = yes)**	−0.779 (0.029)	−1.03 (0.000)	−0.201 (0.404)	−0.254 (0.218)	−0.276 (0.087)	0.219 (0.109)
**Smoking (ref. cat. = never)**						
**Past Smoker**	−0.008 (0.983)	0.425 (0.202)	0.413 (0.106)	0.088 (0.706)	0.013 (0.940)	−0.088 (0.578)
**Current Smoker**	0.486 (0.366)	−0.642 (0.247)	0.607 (0.068)	0.121 (0.767)	0.375 (0.132)	0.175 (0.544)
**Acculturation**	0.513 (0.019)	0.063 (0.804)	−0.179 (0.136)	0.138 (0.489)	0.046 (0.574)	0.057 (0.661)
**El Paso (ref cat. = Cameron County)**	10.34 (0.000)		0.281 (0.122)		0.331 (0.006)	
